# Identification of a novel immunogenic death-associated model for predicting the immune microenvironment in lung adenocarcinoma from single-cell and Bulk transcriptomes

**DOI:** 10.7150/jca.98659

**Published:** 2024-08-13

**Authors:** Xinyu Pan, Huili Chen, Linxiang Zhang, Yiluo Xie, Kai Zhang, Chaoqun Lian, Xiaojing Wang

**Affiliations:** 1 Anhui Province Key Laboratory of Clinical and Preclinical Research in Respiratory Disease, The Department of Pulmonary Critical Care Medicine, First Affiliated Hospital of Bengbu Medical University, Bengbu, 233030, China.; 2 Department of Medical Imaging, Bengbu Medical University, Bengbu 233030, China.; 3 Research Center of Clinical Laboratory Science, Bengbu Medical University, Bengbu, 233030, China.; 4 Department of Clinical Medicine, Bengbu Medical University, Bengbu, 233030, China.; 5 Department of Genetics, School of Life Sciences, Bengbu Medical University, Bengbu, 233030, China.; 6 Molecular Diagnosis Center, Joint Research Center for Regional Diseases of IHM, The First Affiliated Hospital of Bengbu Medical University, Bengbu, 233030, China.

**Keywords:** Lung adenocarcinoma, Immunogenic cell death, Single-cell RNA-seq, Prognosis, Immunotherapy efficacy

## Abstract

**Background:** Studies on immunogenic death (ICD) in lung adenocarcinoma are limited, and this study aimed to determine the function of ICD in LUAD and to construct a novel ICD-based prognostic model to improve immune efficacy in lung adenocarcinoma patients.

**Methods:** The data for lung adenocarcinoma were obtained from the Cancer Genome Atlas (TCGA) database and the National Center for Biotechnology Information (GEO). The single-cell data were obtained from Bischoff P et al. To identify subpopulations, we performed descending clustering using TSNE. We collected sets of genes related to immunogenic death from the literature and identified ICD-related genes through gene set analysis of variance (GSVA) and weighted gene correlation network analysis (WGCNA). Lung adenocarcinoma patients were classified into two types using consistency clustering. The difference between the two types was analyzed to obtain differential genes. An immunogenic death model (ICDRS) was established using LASSO-Cox analysis and compared with lung adenocarcinoma models of other individuals. External validation was performed in the GSE31210 and GSE50081 cohorts. The efficacy of immunotherapy was assessed using the TIDE algorithm and the IMvigor210, GSE78220, and TCIA cohorts. Furthermore, differences in mutational profiles and immune microenvironment between different risk groups were investigated. Subsequently, ROC diagnostic curves and KM survival curves were used to screen ICDRS key regulatory genes. Finally, RT-qPCR was used to verify the differential expression of these genes.

**Results:** Eight ICD genes were found to be highly predictive of LUAD prognosis and significantly correlated with it. Multivariate analysis showed that patients in the low-risk group had a higher overall survival rate than those in the high-risk group, indicating that the model was an independent predictor of LUAD. Additionally, ICDRS demonstrated better predictive ability compared to 11 previously published models. Furthermore, significant differences in biological function and immune cell infiltration were observed in the tumor microenvironment between the high-risk and low-risk groups. It is noteworthy that immunotherapy was also significant in both groups. These findings suggest that the model has good predictive efficacy.

**Conclusions:** The ICD model demonstrated good predictive performance, revealing the tumor microenvironment and providing a new method for evaluating the efficacy of pre-immunization. This offers a new strategy for future treatment of lung adenocarcinoma.

## Introduction

Lung cancer remains one of the deadliest cancers in the world, 85% of which is non-small cell lung cancer (NSCLC) 1. Lung adenocarcinoma (LUAD) is the most common subtype of lung cancer worldwide, accounting for approximately 40% of all lung cancer cases 2, 3. Currently, lung adenocarcinoma (LUAD) is treated with surgery, radiotherapy, chemotherapy, targeted therapy and immunotherapy or a combination of these therapies 4. Although advances on treatment strategies for LUAD has been made, the overall 5-year survival rate is still at a low level with unoptimistic prognosis (less than 20%) 5. This requires the discovery of new therapeutic targets for LUAD and effective combination therapy strategies.

Over the past decade, the Cell Death Nomenclature Committee has defined and interpreted cell death from morphological, biochemical, and functional perspectives 6. Immunogenic cell death (ICD) plays a key role in immune surveillance 7; ICD is designed to stimulate the immune system of an immunocompetent host. When ICD occurs, a large number of damage-associated molecular patterns (DAMP) are exposed and released, providing a powerful adjuvant boost to dying cancer cells by attracting and activating antigen-presenting cells 8, 9. DAMP-mediated ICD involves multiple innate immune receptors, and their collaboration with DAMP is required for ICD and antitumor immune responses 10. DAMP-mediated ICD involves multiple innate immune receptors, and ICD and antitumor immune responses require their collaboration with DAMP. However, the therapeutic potential and mechanisms of utilizing ICD to treat LUAD have not been thoroughly investigated. Therefore, an in-depth understanding of the correlation between ICD-related genes and overall survival in LUAD may provide new approaches for the treatment and prognostic evaluation of LUAD patients.

The objective of this study was to identify ICD-related biomarkers of lung adenocarcinoma (LUAD) that could predict the effectiveness of conventional therapies and suggest the potential for immunotherapy. A single-cell RNA sequencing (scRNA-seq) dataset was used to identify genes related to immunogenic cell death (ICD) in LUAD. The identified genes were used for consistent clustering to classify LUAD into two subtypes, and the differential genes between the two groups were further analyzed. In this instance, we created an ICD prediction model (ICDRS) and compared it with 11 other published models. Additionally, we discuss the immunological characteristics of the population defined by ICDRS. Finally, we found that ICDRS successfully predicted the outcome and success of immunotherapy in LUAD patients. Our analysis showed that ICDRS has good predictive efficacy. Our findings suggest that ICDRS is a prognostic model with good predictive efficacy.

## Materials and Methods

### Data collection and processing

Transcriptomic data of LUAD patients and corresponding clinical data were obtained from the TCGA (https://portal.gdc.cancer.gov/) database and GEO (http://www.ncbi.nlm.nih.gov/geo/) database. 500 LUAD cases (primary tumor samples with complete survival information) from the TCGA database were used to construct relevant prognostic signatures, and GSE31210 (n=226) and GSE50081 (n=127) were used to validate the centrosome-related genes for centrosomal prognosis based on LUAD Related Characteristics. In order to identify ICD-related genes, we collected 32 genes from previously reported literature 11, 12 (**Supplementary Table 1**).

### Processing of single-cell data

The scRNA-seq dataset for lung adenocarcinoma was obtained from the article 'Single-cell RNA sequencing reverses distinct tumor microenvironmental patterns in lung adenocarcinoma'^13^. Initially, the term 'Single-cell RNA sequencing' was used. The 10×scRNA-seq data was converted into Seurat objects using the 'Seurat' R package. Cells with substandard quality were excluded, and quality control (QC) was performed by calculating the percentage of mitochondrial or ribosomal genes 14. We identified highly variable genes for subsequent analysis and used the 'Harmony' tool to remove batch effects. Cell clusters were constructed using the 'FindClusters' and 'FindNeighbors' functions and visualized with the 't-SNE' method. Cellular annotation was performed based on marker genes for different cell types. The Seurat package's 'AddModuleScore' function was used to quantify the activity of a specific set of genes in each cell. To analyze the differentially expressed genes (DEGs) between the two groups, the Seurat package's 'FindMarkers' function was used. The statistical significance of differentially expressed genes (DEGs) was calculated using the Wilcoxon test (p.adj < 0.05), while genes differentially expressed between cells with high and low ICD scores at the single-cell transcriptome level were considered to be involved in ICD. All other parameters were set to default values. These genes were subsequently included in the overall transcriptome level analysis of WGCNA.

### Identification of immunogenic death-related genes

WGCNA (Weighted Correlation Network Analysis) is a systems biology approach for identifying patterns of genetic relationships between samples. WGCNA can be used to find highly synergistic genomes and to search for potential biomarker genes or therapeutic targets based on the endogenous nature of the genome and the linkage between the genome and the phenotype 15. WGCNA can be used to search for highly synergistic genomes and to find potential biomarker genes or therapeutic targets based on their endogenous nature and their association with phenotype. Using "limma" 16 Analyze differential genes. Intersect the differential genes with the genes identified by WGCNA, i.e., immunogenic death-related genes (ICDRGs).

### Consensus clustering of immunogenic death-related genes

In this study, we screened for prognostically relevant immunogenic death-associated genes and used prognostically relevant immunogenic death-associated genes. We used the "ConsensusClusterPlus" package for consistent clustering, and the optimal number of clusters was assessed by the cumulative distribution function (CDF) plot and the consensus heatmap with an optimal K-value of 2. We used the "survival" package for the assessment of LUAD samples in molecular subtypes based on ICDRGs. We evaluated the clinical survival outcomes of LUAD samples in molecular subtypes based on ICDRGs. Finally, we used the "pheatmap" R package to visualize the relationship between ICDRGs expression, clinical survival status and clinicopathological features.

### Enrichment analysis and functional annotation

To further investigate differentially expressed genes (DEGs) between subgroups defined by ICDRGs, we used the "limma" R package, where genes associated with prognosis were further analyzed (|logFc|> 1 & p < 0.05). To explore the underlying mechanisms of the two immunogenic death subtypes involved in LUAD, we performed gene set enrichment analysis (GSEA) in different clusters constructed based on immunogenic death-related genes. The "h.all.v7.4.Hs.symbols" and "c2.cp.kegg.v7.4.symbols.gmt" gene sets downloaded from MsigDB were used as reference gene sets, and we used the "GSVA" gene set as the reference gene set. "GSVA" package to calculate the enrichment scores of the relevant pathways. We calculated the differentially expressed pathways between the two subgroups, where P < 0.05 was considered significant. The gene set of GSVA was downloaded from Molecular Signatures Database (MSigDB) v7.4 database 17.

### Construction and validation of ICD-related prognostic risk profiles

To explore the prognostic value of ICDRGs based on LUAD, we performed one-way Cox regression analysis (P < 0.05) and least absolute shrinkage and selection operator regression (LASSO) analysis of DEGs between subgroups 18 that identified independent characteristic prognostic factors in order to establish the prognostic profile of LAUD. An immunogenic mortality risk score (ICDRS) was then calculated for each LUAD patient based on the risk coefficients and LUAD expression profiles obtained in the LASSO regression analysis, using the formula: ICDRS = 0.064*TPX2+-0.034*SFTPB+0.069*RHOV+-0.051*SERPIND1+-0,063* FDCSP+0.106*FAM83A+ 0.031*CPS1+0.063*KRT6A. Subsequently, we used the LUAD patients from the TCGA cohort as the training set and GSE31210 and GSE50081 from the GEO database as the validation set, and categorized the LUAD patients into two groups, the low-risk and the high-risk groups, based on the median of the risk score. Kaplan-Meier survival curves and Log-Rank tests were used to assess whether there was a significant difference in OS between the low-risk and high-risk groups. Finally, we validated the prognostic predictions of the risk model by calculating 1-, 3-, and 5-year AUC values in the validation cohort using time-dependent ROC curves.

### Characterization of the LUAD immune profile

The 'Estimate' algorithm was used to calculate immune microenvironment (TME) scores between the high- and low-risk groups 19. The relative proportions of the 22 immune cell types in each tumor tissue were estimated using the CIBERSORT algorithm based on the TPM values of the TCGA-LUAD patients, and samples with P>0.05 in the results were excluded and the remaining samples were further analyzed 20. In addition, we determined the level of immune cell infiltration in LUAD TME by using the single sample gene set enrichment analysis (ssGSEA) algorithm 21 and unique combinations of characterized genes for each immune cell subtype were obtained from the most recent literature 22, 23 The unique combinations of genes for each immune cell subtype were obtained from the most recent literature.

### Immunotherapy prediction

We collected three GEO immunotherapy cohorts (GSE78220 24) and the IMvigor210 cohort to study the correlation between ICD characteristics and immunotherapy. We processed the data using the "IMvigor210CoreBiologies" R package from the IMvigor210 cohort 25. We used the "IMvigor210CoreBiologies" R package from the IMvigor210 cohort to process data. In addition, to determine immunogenicity based on immunomodulators, immunosuppressive cells, MHC molecules, and effector cells, we used the Immunophenoscore (IPS) algorithm, which calculates the IPS score based on the unbiased gene expression of a representative cell type using a machine-learning methodology. Higher IPS scores are indicative of a better response to immunotherapy. IPS scores for TCGA-LUAD patient samples were obtained from The Cancer Immunome Atlas (TCIA) database (https://tcia.at/home).

### Cell line culture and RT-qPCR

All cells were cultured at 37°C in an incubator with 5% CO_2_ atmosphere. Normal human lung cell line BEAS-2B, lung adenocarcinoma cells NCI-H1299 and A549 were obtained from the Chinese Academy of Sciences (Shanghai, China). Cell culture media, plates and dishes were from Thermo Fisher Scientific (Invitrogen, USA) and Corning Inc. BEAS-2B cells, NCI-H1299 cells and A549 cells were detached and inoculated into 60 mm culture dishes overnight at an initial density of 1×10^6^ cells/well. Subsequently, SYBR Green qPCR mix (Vazyme, China) was used to synthesize cDNA for real-time PCR. Our results were analyzed using the comparative Ct method and the Ct values of each gene were normalized by the Ct reads of the corresponding GAPDH. All data are expressed as mean ± standard deviation (SD) of three independent experiments.

### Statistical analysis

All statistical analyses were performed using R software (version 4.2.2). Wilcoxon test was used to compare the differences between groups. The log-rank test was used to compare Kaplan-Meier survival curves. Univariate and multivariate Cox analyses were performed to establish independent prognostic factors. All P values were two-sided and less than 0.05% were considered statistically significant. All P values were two-sided and less than 0.05 were considered statistically significant.

## Results

### Identification of ICD-related genes from single-cell transcriptomes

**Figure 1:**
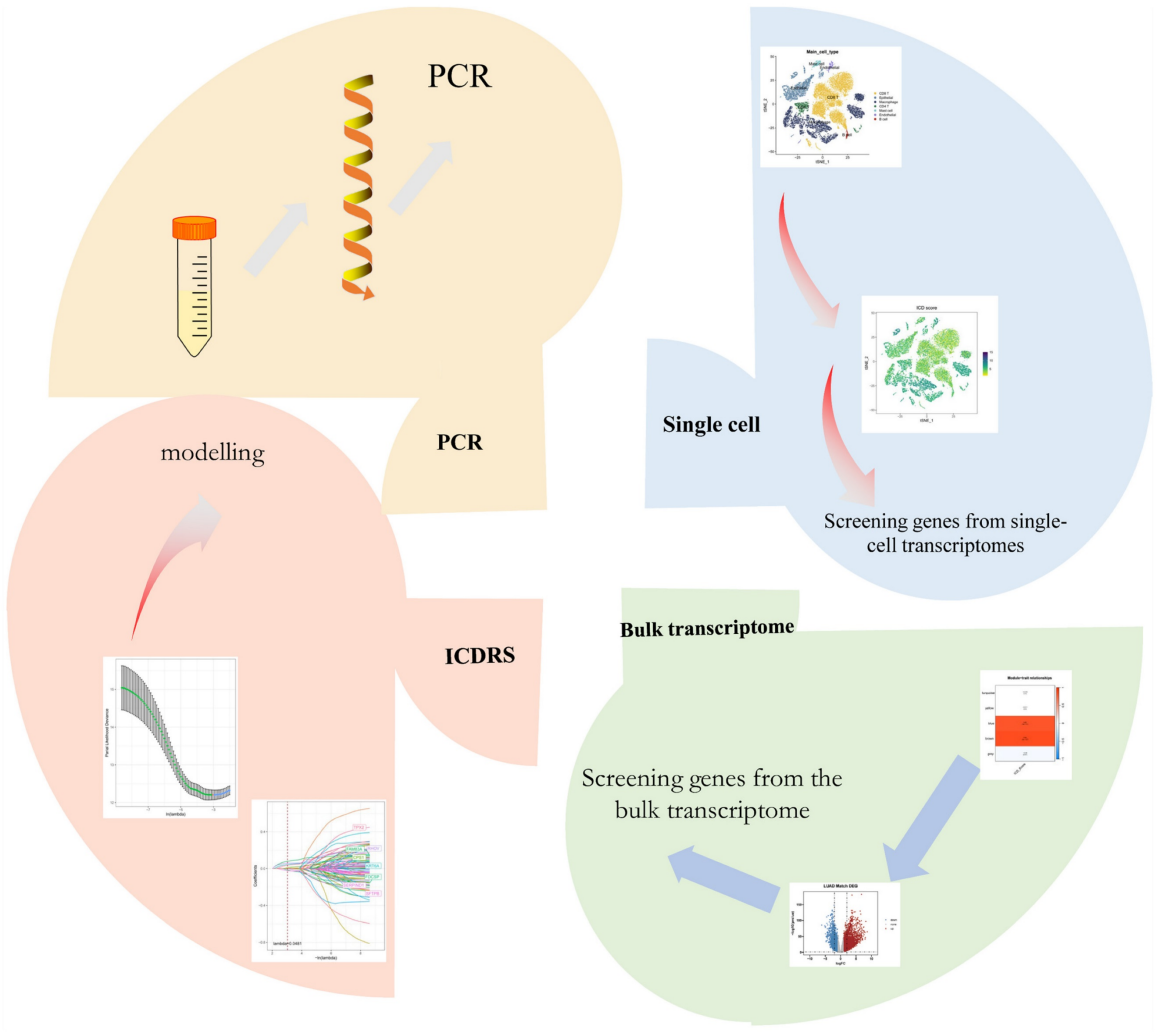
Flowchart of this study.

**Figure 2:**
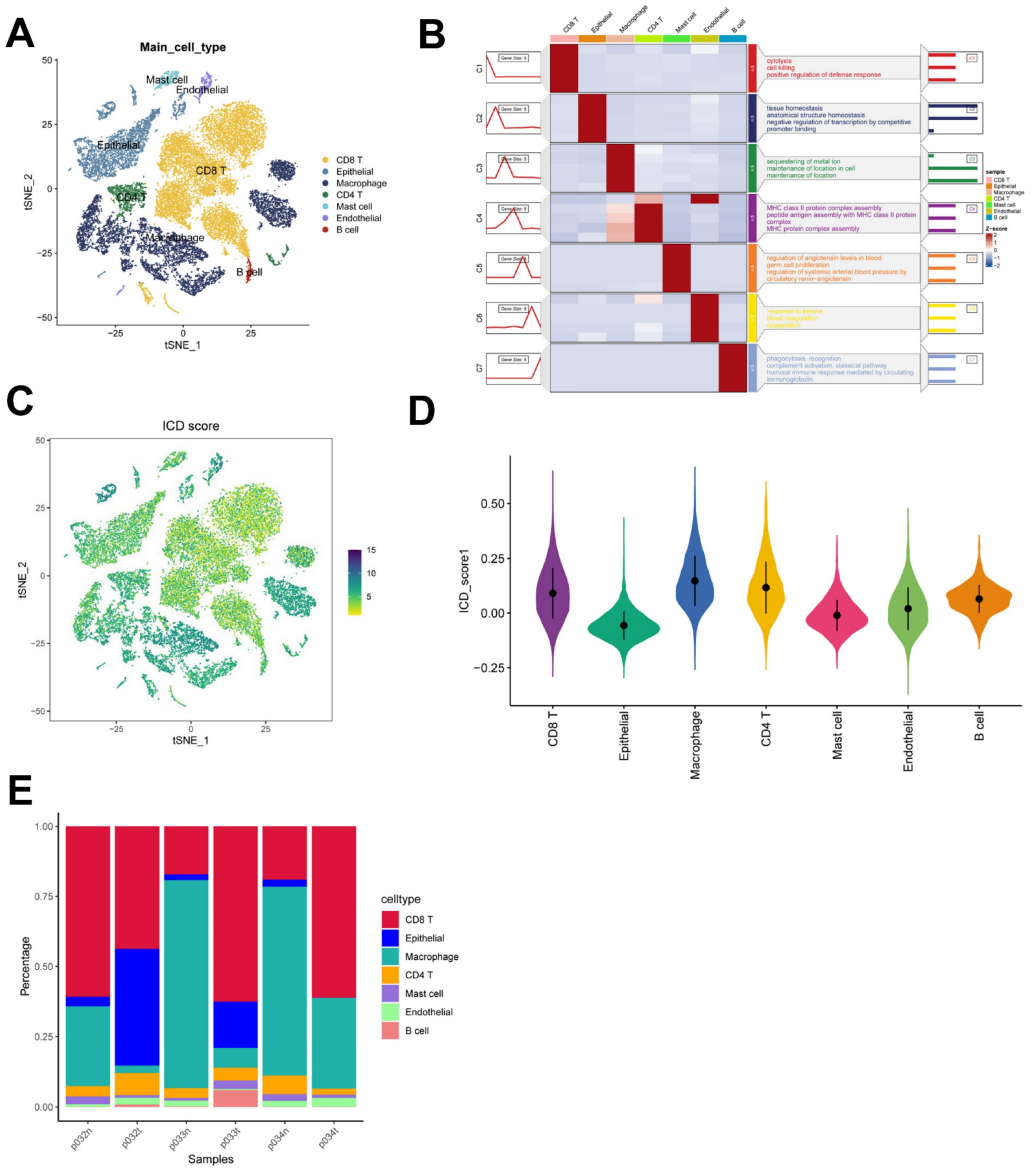
** identification of ICD-related genes from single-cell transcriptomes. (A)** t-SNE plot showing cell types recognized by marker genes.** (B)** Heatmap showing the five most important marker genes in each cell cluster**. (C)** Immunogenic death score (ICD) for each cell.** (D)** Distribution of ICD scores in different cell types.** (E)** Cell scale diagram.

The workflow of our study is shown in Figure 1. Initially, we collected a collection containing 32 immunogenic death genes from the literature and databases. Subsequently, we used these 32 genes to score the single-cell transcriptome, as shown in Figure 2C. We then performed differential analysis on the high and low groups, and the differential genes obtained were incorporated into the WGCNA, and the genes obtained from the WGCNA were intersected with the differential genes to take the intersection, which resulted in 167 genes, and we subsequently used a one-way cox screen to obtain 40 genes related to OS, and we used these 40 genes to perform a consistency clustering, which resulted in the classification of TCGA-LUAD into two subtypes. These two subtypes were analyzed for differences and screened using a one-way cox screen and included in a LASSO regression analysis, which was ultimately performed by 8 genes to establish an immunogenic death-related model (ICDRS). Immunogenic death-related modeling (ICDRS) was performed for these 8 genes and compared with published article models, which showed good prognostic efficacy for ICDRS. We investigated the survival analysis, pathway enrichment analysis, immune infiltration profile and immunotherapy analysis of ICDRS and screened three genes by ROC diagnostic curve for validation by RT-qPCR.

We used the 6 single-cell dataset with 27,066 cells after filtering from Philip Bischoff et al. After descending clustering using TSNE analysis, we annotated cell subpopulations and identified seven cell types including macrophages, epithelial cells, endothelial cells, CD4 T cells, CD8 T cells, mast cells, and B cells (Figure 2 A). The heatmap shows the top five marker genes for each cell population (Figure 2 B). To quantify the activity of immunogenic death (ICD) in different cell types, we used the "AddModuleScore" function in the Seurat software package to calculate the expression levels of the set of ICD-related genes in all cells (Figure 2C). Among the seven cell types, we observed significantly elevated ICD activity in macrophages, CD4 T cells, and endothelial cells (Figure 2D). Based on the ICD activity, we classified the cells into high ICD and low ICD groups and identified 1291 differentially - expressed genes (DEGs) between the two groups for further analysis (**Supplementary Table 2**). Figure 2E presents the proportion of each cell type in the 6 samples.

### Identification of ICD-related genes in the bulk-RNA-seq transcriptome

**Figure 3:**
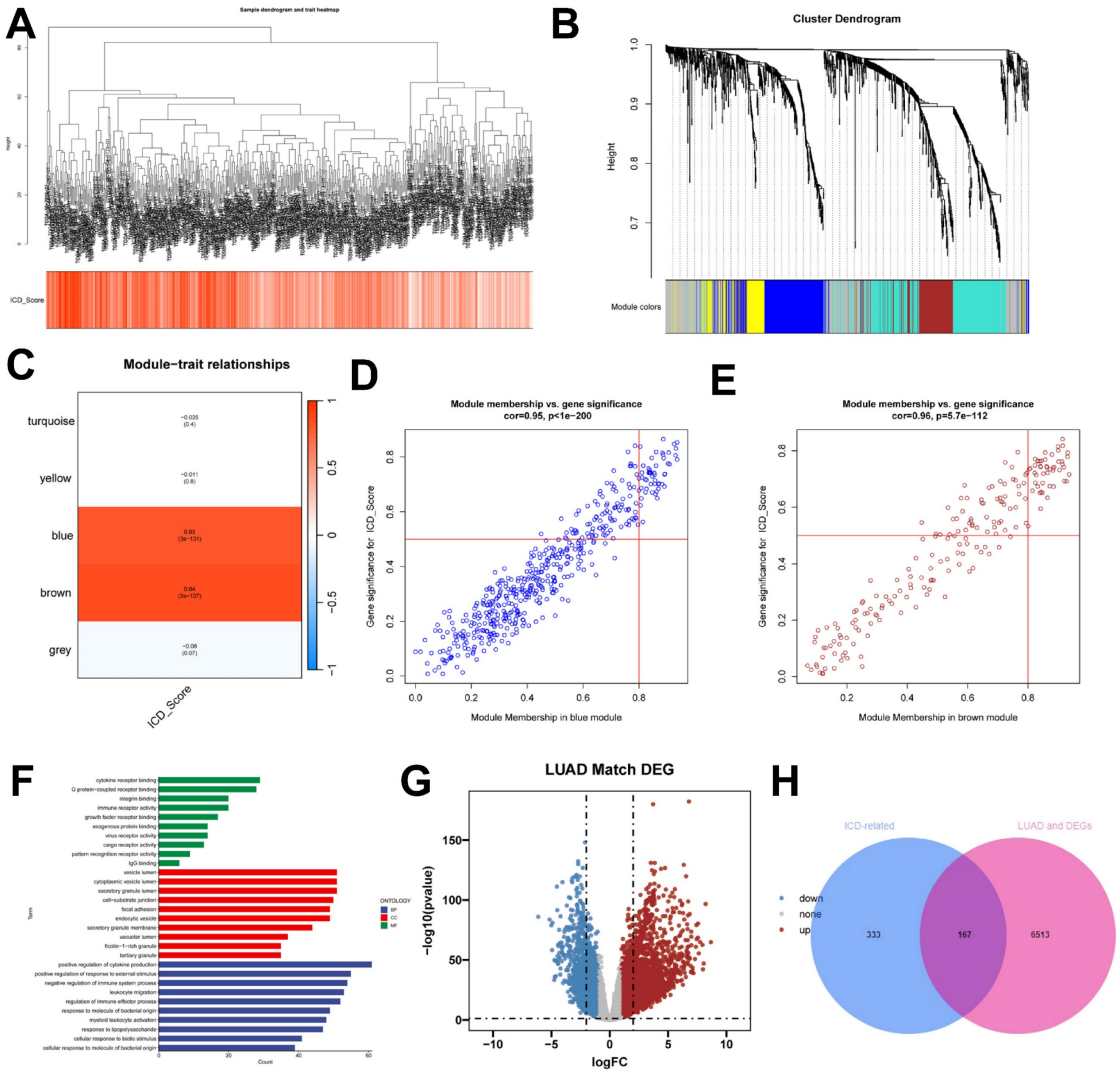
** identification of ICD-related genes from the bulk transcriptome. (A)** Dendrogram showing hierarchical clustering of TCGA-LUAD samples, with the heatmap at the bottom indicating the ICD score for each sample, as calculated by the ssGSEA algorithm.** (B)** Clustering dendrogram analysis of WGCNA.** (C)** Heatmap of module features showing that the MEbrown and MEblue modules are closely associated with ICD features.** (D, E)** Scatter plots showing the relationship between gene significance (GS) and module membership (MM) in brown and blue modules.** (F)** GO enrichment analysis of genes** (G)** Volcano plot showing the results of differential analysis of TCGA-LUAD tumor samples versus normal samples.** (H)** Venn diagram showing crossover genes between MEbrown and MEblue modules and DEGs in bulk-RNA-seq.

In this study, we utilized the ssGSEA algorithm to obtain ICD activity scores for each TCGA-LUAD sample, which served as phenotypic data for subsequent WGCNA analysis. To identify modules significantly correlated with ICD scores, we applied WGCNA analysis to the TCGA-LUAD dataset. After removing outlier samples, a co-expression network was constructed using 1291 DEGs identified at the single-cell-seq level (Figure 3A). To ensure that the topological network was scale-free, the optimal soft threshold for power = 4 was chosen (**Supplementary Figure 1D**). By setting the minimum module gene count to 60 and medissres to 0.25, a total of 5 modules were obtained (Figure 3B). Our results showed that MEbrown and MEblue modules were closely associated with ICD scores in bulk-RNA-seq (Figure 3C). In addition, scatter plots of gene significance (GS) versus module membership (MM) for the brown and blue modules showed a significant correlation (Figure 3D, E), suggesting that the genes in these two modules may have functional significance related to immunogenic death. The genes in these two modules were analyzed by GO enrichment (Figure 3F). The volcano plot (Figure 3G) shows the differentially expressed genes (DEGs) between tumor and normal lung tissues in TCGA-LUAD bulk-RNA-seq (|logFC|> 1 and p.d adj < 0.05). We crossed 500 genes from these two modules with DEGs from bulk-RNA-seq and finalized 167 genes (Figure 3H). These genes were named Immunogenic cell death -related genes (ICDRGs) and are thought to be involved in ICD at the bulk-RNA-seq and single-cell transcriptome levels.

### Recognition of ICD-associated clusters and altered biological processes

**Figure 4:**
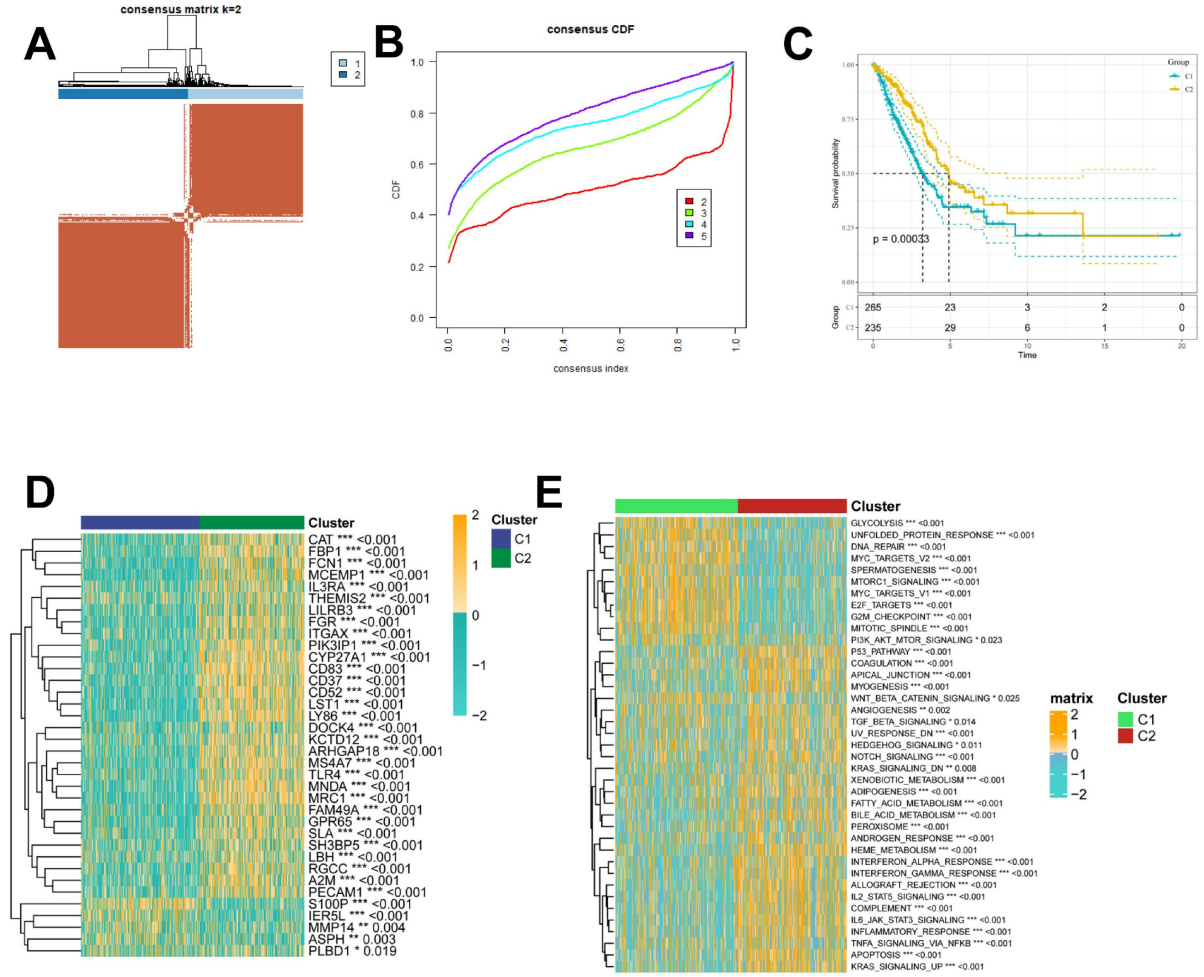
** Identification of ICD molecular subtypes. (A)** Consensus heatmap matrix and correlation region for two clusters (k = 2)** (B)** indicates that clustering results are best at K = 2.** (C)** Survival analysis indicates that C2 has a better prognosis.** (D)** Difference in ICD expression levels between the two subtypes, p < 0.05.** (E)** GSVA demonstrates the HALLMARK pathway for the different subtypes, with yellow color representing promotion and blue color representing inhibition.

To further explore the profile and characterization of immunogenic death-related genes in LUAD, this study applied a consensus clustering algorithm to classify LUAD patients based on the expression of ICDs associated with OS. To obtain the optimal number of clusters (k-value), we calculated the consistency coefficient and found that k = 2 was the best choice for classifying the entire cohort into clusters C1 (n = 265) and C2 (n = 235) (Figure 4A, B). Kaplan-Meier survival analysis showed that C2 had superior OS in LUAD (p < 0.0001) (Figure 4C). In addition, we obtained consistent results on the GSE31210 cohort **(Supplementary Figure 1A-C)**, and we compared the clinicopathologic features and expression of immunogenic death-associated genes in the two subtypes. Some ICDs were highly expressed in C2, such as CAT, FBP1, FCN1, and FGR, while some ICDRGs, including S100P, IER5L, MMP14, and PLBD1, were highly expressed in C1 (Figure 4D). The different pathway relationships between the two subtypes were next compared. We performed a GSVA based on the tumor Hallmark gene set to investigate the molecular biological functions of TRP isoforms, and the heatmap demonstrated the pathways with significant differences. The results showed that C1 was significantly enriched in pathways significantly associated with oncogenic activation and highly proliferative features, such as MYC target V1/V2, G2M checkpoint, E2F target PI3K/AKT/mTOR, unfolded protein response, glycolysis, and DNA repair. And C2 is highly expressed in immune pathways, such as IL2/STAT5 signaling pathway, IL6/JAK/STAT3 signaling pathway, allograft rejection, inflammatory response. Also, oncogenic pathways, such as TGFβ signaling pathway, NOTCH signaling pathway and Hedgehog signaling pathway were highly enriched in C2 (Figure 4E).

### Construction and validation of ICD-related prognostic features

**Figure 5:**
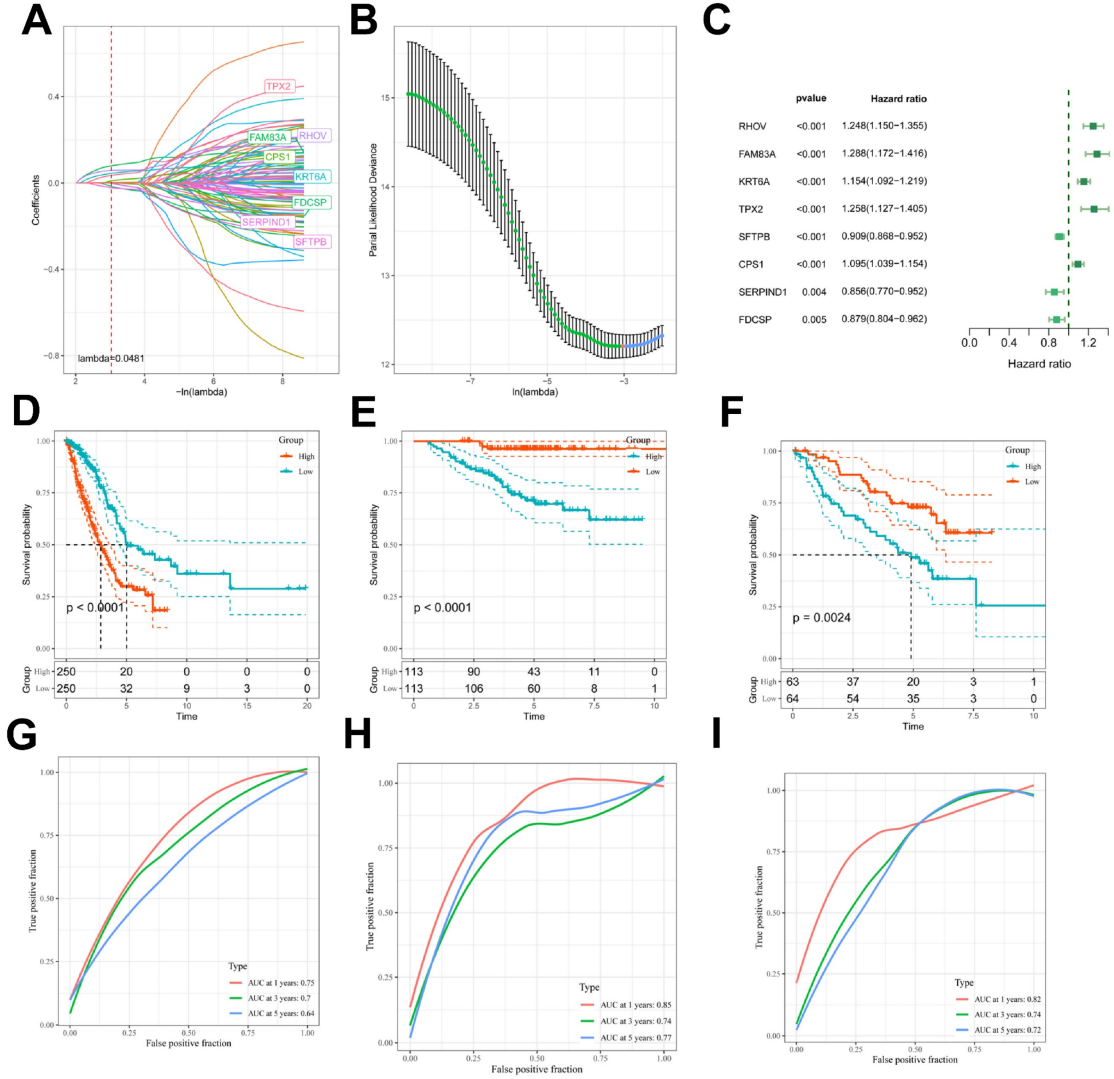
** Construction and validation of ICD-related prognostic features. (A)** Ten-fold cross-validation of parameter selection adjusted by LASSO regression.** (B)** Screening of coefficients under LASSO analysis. A vertical line is plotted at the value selected by 10-fold cross-validation of overall survival.** (C)** Forest plot showing univariate Cox results.** (D-F)** KM curves comparing time-dependent ROC curve analyses in the TCGA-LUAD** (D)**, GSE31210** (E)**, and GSE50081** (F)** high and low risk groups of patients with LUAD** (G-I)** in the TCGA-LUAD** (G)**, GSE31210** (H)**, and GSE50081** (I)** cohorts.

Next, we also performed inter-subgroup DEGs, and to further assess the impact of DEGs on survival prognosis, we first used univariate Cox regression analysis (p < 0.05) and found that 100 genes were significantly associated with OS (**Supplementary Table 3**). Next, a 10-fold cross-validated LASSO regression analysis was performed on these 100 genes, and 8 genes (FAM83A,RHOV,CPS1,TPX2,SFTPB, SERPIND1, FDCSP, KRT6A) were screened for further analysis, and the Immunogenic Death Related Risk Score (ICDRS) for each LUAD patient was based on the following equation Calculated as ICDRS= 0.064*TPX2+-0.034*SFTPB+0.069*RHOV+-0.051*SERPIND1+-0,063*FDCSP+0.106*FAM83A+ 0.031*CPS1+0.063*KRT6A (Figure 5A, 5B), and the forest plot in Figure 5C illustrates the eight gene cox results. We assigned LUAD patients to either the high-risk or low-risk group based on the median risk score. Kaplan-Meier analysis showed that patients in the high-risk group had worse OS (p<0.0001; Figure 5D-F), and the analysis of subject work characteristics (ROC) curves showed that the area under the curve (AUC) of the ICDRS in the TCGA training set at 1 year, 3 years, and 5 years respectively reached 0.75, 0.7, and 0.64, indicating good prognostic diagnostic efficacy, and the validation set GSE31210 was 0.85,0.74, and 0.77. To further validate the accuracy and reliability of the eight-gene model, we performed additional validation in the GSE50081 cohort, and the validation cohort GSE50081 was 0.82, 0.74, and 0.72, the ICD-related prognostic features showed superior performance (Figure 5G-I). In addition, we obtained clinical information for the high ICDRS and low ICDRS groups (Table 1).

### Comparison of ICDRS with other published articles

To compare the prognostic efficacy of ICDRS with existing LUAD models, we integrated 11 previous studies that used different biologically meaningful features such as arginine-substituted succinate 26, copper death 27, necrotic apoptosis 28, immune activation 29 ubiquitin proteasome 30 and autophagy 31. Notably, ICDRS exhibited better C-index performance than almost all models in the TCGA-LUAD, GSE31210, and GSE50081 datasets (Figure 5A-C). Altogether, these findings confirm the idea that ICDRS is a more effective prognostic model for LUAD.

### Clinical Relevance and Survival Analysis of ICDRS in Patients with LUAD

**Figure 6:**
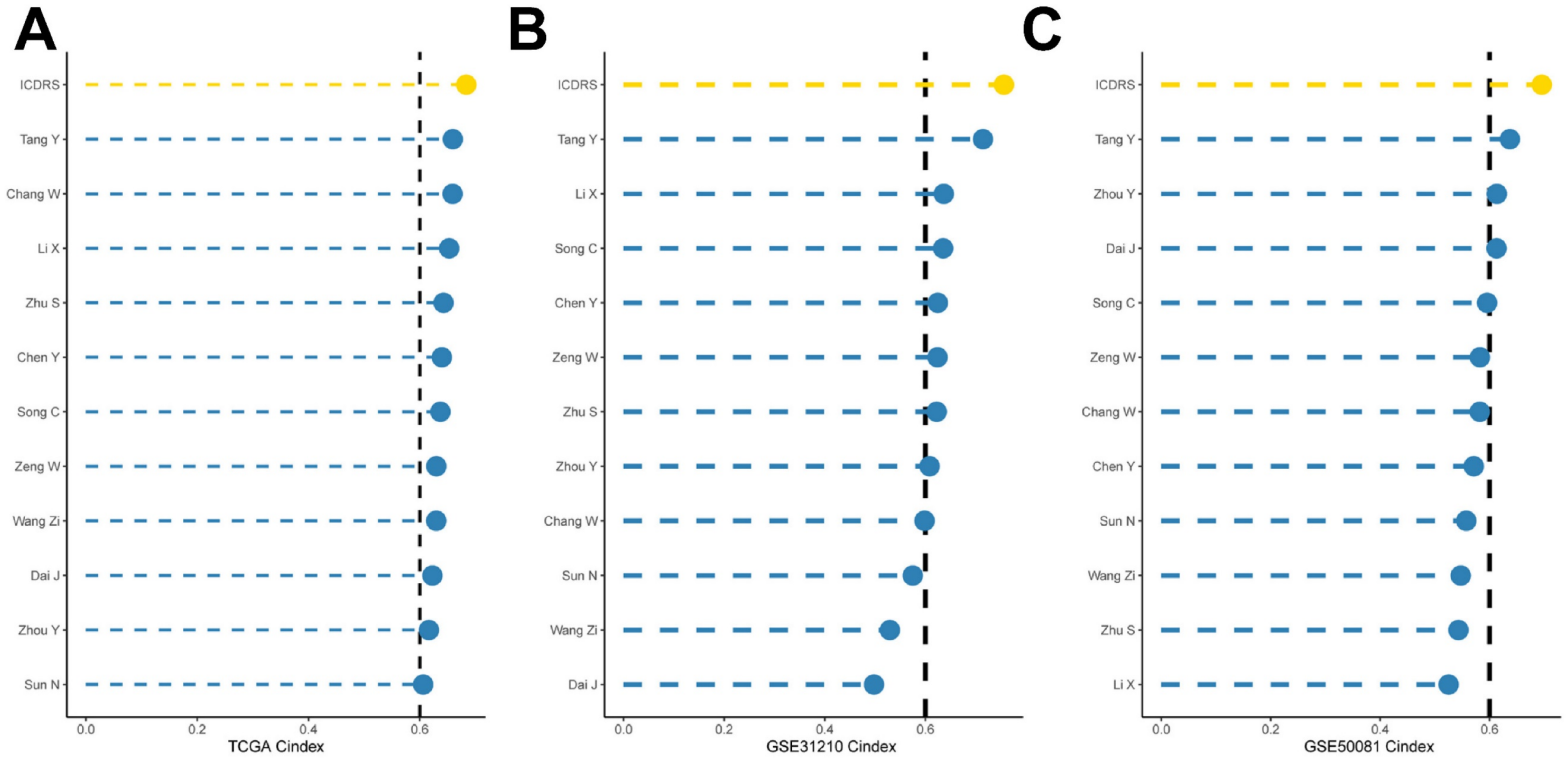
** Comparison of the predictive effect of ICDRS with existing features. (A-C)** Comparison between ICDRS and the other 11 published models in the TCGA-LUAD, GSE31210 and GSE50081 cohorts.

Given the significant differences in individual clinical characteristics of OS between the high and low ICDRS groups, in order to explore and compare such differences more specifically, we categorized LUAD patients into six different subgroups based on clinical characteristics. These were age, pathologic Stage (I-II and III-IV), gender (female and male), pathologic M-stage (M0-1), N-stage (N0-N1) and T-stage (T1-2 and T3-4). Notably, in all subgroups, patients in the low ICDRS group had a significant survival advantage in terms of longer survival time compared with patients in the high ICDRS group (Figure 6A-G, **Supplementary Figure 1E**). Based on the analysis of the results, we are more convinced that the ICD model is a reliable clinical prediction tool.

### Gene set enrichment analysis

**Figure 7:**
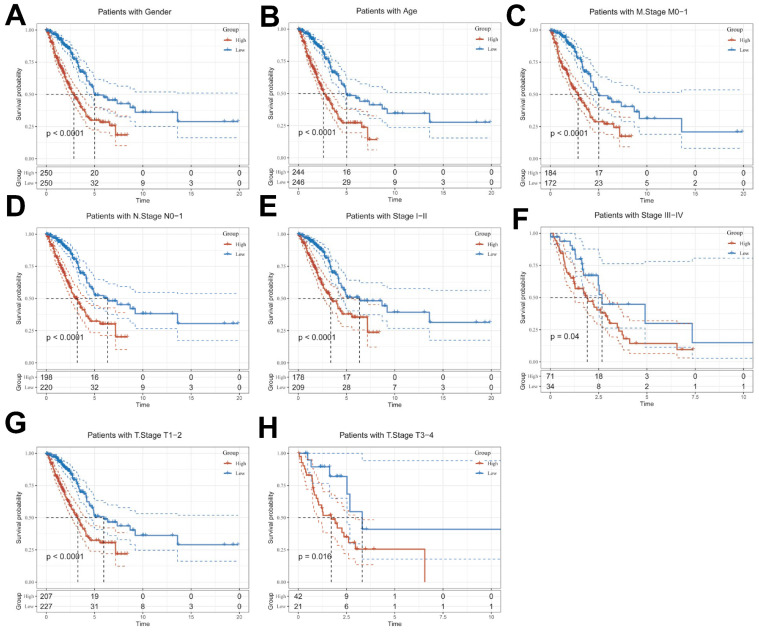
** Clinical relevance and survival analysis of ICDRS in LUAD patients. (A)** Gender.** (B)** Age.** (C)** Pathologic M stage.** (D)** N staging.** (E)** Total staging (I-II).** (F)** Total staging (III-IV).** (G)** T-staging (T1-2).** (H)** T staging (T3-4).

GSEA was used to identify KEGG gene sets enriched in both ICDRS groups. The gene set of the low ICDRS group was enriched for immune-related pathways such as B Cell Receptor Signaling Pathway, Intestinual Immune Network for IGA Production, etc. whereas the gene set of the high ICDRS group was enriched for cell cycle- and cancer-related pathways (Figure 7B, C). GSVA analyzed the differentially enriched HALLMARK pathways between the two groups (Figure 7A). The results showed that the high-risk group was predominantly enriched to oncogenic pathways, while the low-risk group was predominantly enriched to immune-related pathways. The ridge plot demonstrated the GO-enriched analyzed pathways between the two groups (Figure 7D). correlation analysis between ICDRS and hallmarks pathway score further supported these findings (Figure 7E), suggesting that ICDRS is closely associated with cancer-related biological processes and metabolic pathways.

### ICD risk score predicts tumor microenvironment and immune cell infiltration

**Figure 9:**
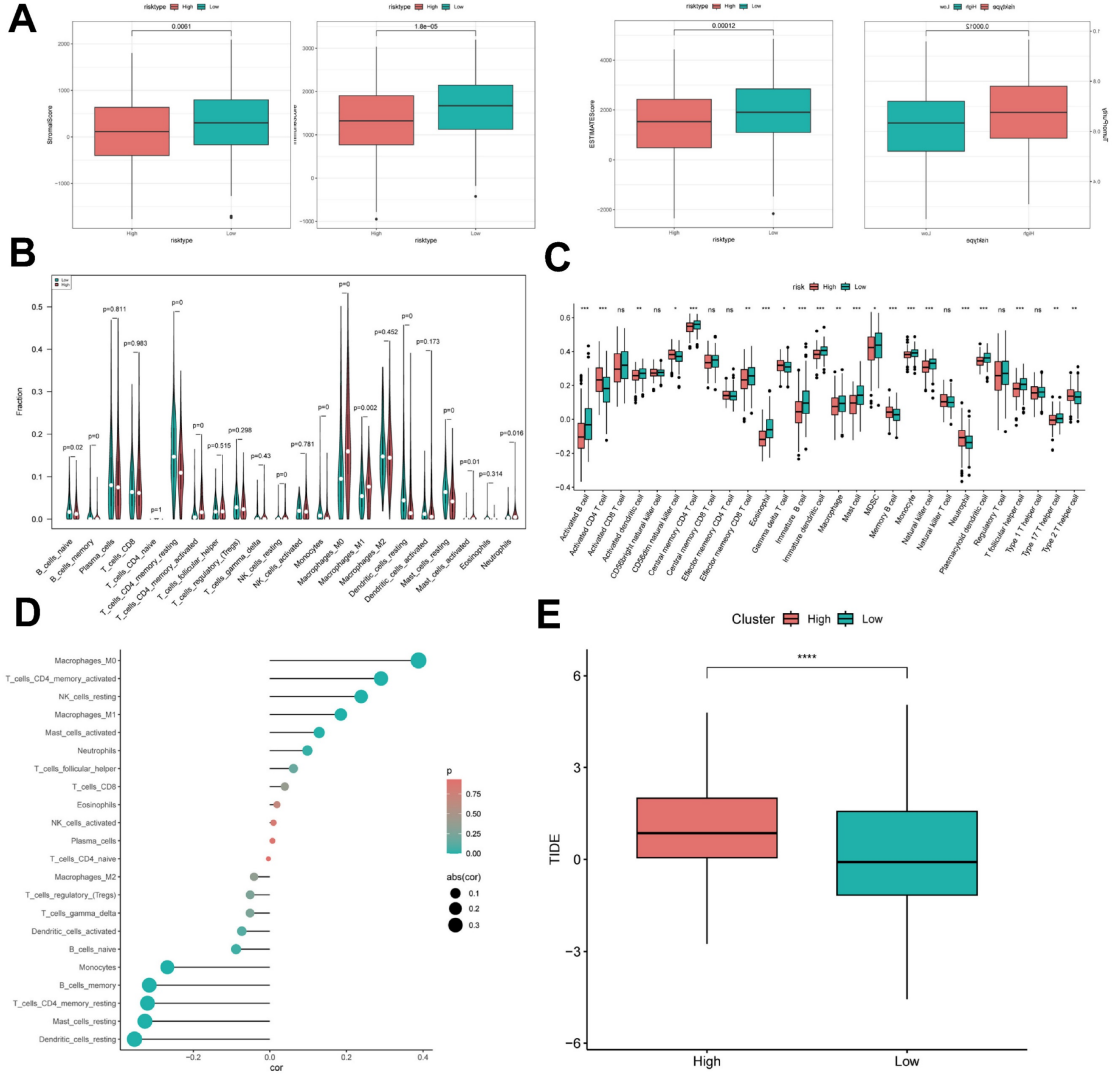
** ICD risk score predicts tumor microenvironment and immune cell infiltration. (A)** Stroma score, immunity score, ESTIMATE score, and tumor purity were used to quantify different immune statuses between high and low ICDRS groups.** (B)** The abundance of each TME-infiltrating cell type was quantified by the CIBESORT algorithm and the ssGSEA algorithm.** (C)** between high and low risk groups.** (D)** Correlation analysis of TME-infiltrating cells with ICDRS.** (E)** Box line plot of TIDE between the two groups.

It has been established that interactions between cancer cells and TME are critical for tumor progression and dissemination 32. Therefore, in this study to assess the immune infiltration status of LUAD samples, we used the ESTIMATE algorithm to calculate the stromal score, immune score, ESTIMATE score, and tumor purity for the ICDRS risk subgroup. The immunity score and ESTIMATE score were significantly higher in the low-risk group, while the tumor purity was higher in the high-risk group (Figure 9A). To further analyze the differences in specific immune cell infiltration between the high- and low-risk groups, we quantified the abundance of immune cell infiltration in each sample using the CIBERSORT algorithm (Figure 9B). The results revealed that Mast cells resting, Dendritic cells resting, and T cells CD4 memory resting were highly expressed in the low ICDRS group, whereas M0 Macrophage and M1 Macrophage were highly expressed in high ICDRS. Next, we used the CIBERSORT results to screen the immune cell types significantly associated with ICDRS by Spearman's correlation analysis (Figure 9D). Similar results were obtained by applying the ssGSEA algorithm for validation (Figure 9C). In addition, the TIDE algorithm was also used, and the results showed that the high ICDRS group had stronger immune escape (Figure 9E).

### Predicting and validating the efficacy of immunotherapy

**Figure 10:**
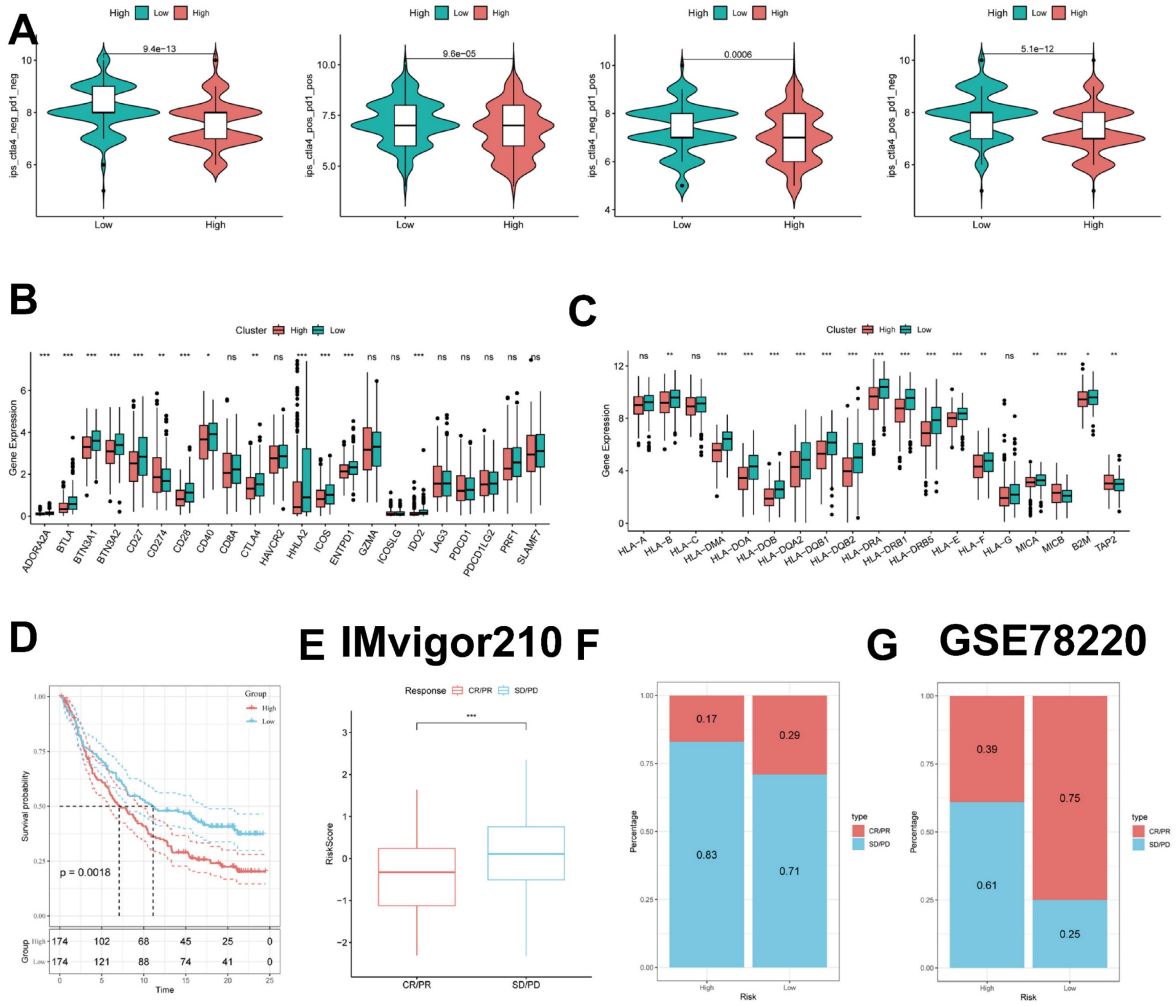
** Predicting and validating the efficacy of immunotherapy. (A)** IPS scores in the high- and low-risk groups.** (B)** Differential expression of various immune checkpoints in the high- and low-risk groups.** (C)** Differential expression of HLA molecules in the high- and low-risk groups.** (D)** Survival curves of the high ICDRS and low ICDRS groups in the IMvigor210 cohort.** (E-F)** Box line plots depicting the difference in risk scores between CR/PR patients and SD/PD patients and the proportion of patients with CR/PR or SD/PD receiving immunotherapy in the IMvigor210 cohort.** (G)** Proportion of patients with CR/PR or SD/PD receiving immunotherapy in the high and low risk groups of the GSE78220 cohort.

To further validate our results, we analyzed the IPS scores obtained from the TCIA database. Higher IPS scores predicted a better response to ICI therapy, including PD-1 inhibitor and CTLA4 inhibitor therapy, and were categorized into four categories: ips_ctla4_pos_pd1_pos, ips_ctla4_pos_pd1_neg, ips _ctla4_neg_pd1_pos, and ips_ctla4_neg_pd1_neg. Our results showed that all four categories were significantly elevated in the low-risk group, suggesting that patients in the low-risk group responded better than patients in the high-risk group to anti-CTLA4 therapy as well as to the combination of anti-pd -1 and anti-CTLA4 therapy (Figure 10B).

Previous studies have reported that high expression of immune checkpoints is associated with better response to immune checkpoint inhibitor (ICI) therapy 33-35. Therefore, we analyzed the differences in immune checkpoints on the basis of risk scores and found that the expression was higher in the low-risk group (Figure 10C). HLA was also strongly associated with immunotherapy 36. We also compared the molecular differences in HLA between the different groups (Figure 10D). In addition, to test the potential of risk scores in predicting immunotherapy in a real immunotherapy cohort, we selected two groups of patients receiving immunotherapy (IMvigor210 and GSE78220) and showed that the proportion of complete response/partial response (CR/PR) was significantly higher and the proportion of response to immunotherapy was significantly higher in the low-risk group (Figure 10E-G). Similarly, in the IMvigor210 cohort, patients at lower risk may have a better prognosis (Figure 10D, **Supplementary Figure 2A-D**). All these results imply that the low-risk group has a favorable immunotherapy effect.

### Identification of key regulatory genes in ICD models

In order to identify the key regulators in the ICD risk subgroups, first we verified the mRNA expression levels of these eight genes, and we found that only SFTPB and SERPIND1 were highly expressed in normal tissues, whereas FAM83A, FDCSP, KRT6A, RHOV and TPX2 were highly expressed in tumor tissues (Figure 10A). In addition, we used ROC diagnostic curves to screen for key regulators, and we found that the only ones with ROC>0.85 were TPX2, RHOV, and FAM83A, and thus we concluded that these three genes were the key regulatory genes in ICDRS (Figure 10B-D, **Supplementary Figure 3A-E**). We also plotted KM curves to verify the survival of these genes (Figure 10E-G, **Supplementary Figure 4A-E**).

These results showed that TPX2, RHOV, and FAM83A were the key regulatory genes in ICDRS. Finally, we evaluated the expression of three core genes in ICDRS in three cell lines, including one normal lung cell line (BEAS-2B) and three lung adenocarcinoma cell lines (A549 and H1299) (Figure 10H-J). The results showed that TPX2, RHOV and FAM83A expression was significantly upregulated in the tumor cell lines.

## Discussion

Despite significant efforts to develop comprehensive treatment strategies, the prognosis for patients with LUAD remains poor, with a 5-year survival rate of 15 percent 37. NSCLC is the most common form of lung cancer pathologic classification. Specifically, LUAD is the main subtype of NSCLC and the most common primary lung cancer 38. LUAD is the main subtype of NSCLC and the most common primary lung cancer. Although lung cancer treatment is being explored and researched, the lack of reliable early prognostic indicators hampers treatment outcomes 39. The lack of reliable early prognostic indicators hinders the therapeutic efficacy. Therefore, we need to explore new biomarkers to better treat patients with lung adenocarcinoma.

The concept of immunogenic cell death has been described as a unique type of regulatory cell death capable of triggering an intact antigen-specific adaptive immune response by emitting a danger signal or DAMP 40-42. The combination of immunogenic therapies and novel immunotherapeutic regimens holds great promise for the treatment of malignant tumors 43-46. The combination of immunogenic therapy and novel immunotherapeutic options holds great promise for the treatment of malignant tumors. Therefore, it may be advantageous to identify ICD-related biomarkers that can help differentiate patients with LUAD, provided they benefit from immunotherapy. However, little is known about the role of immunogenic death in LUAD.

We obtained immunogenic death-related genes from single-cell transcriptomes using scRNA-seq data from the article by Philip Bischoff et al. The TCGA-LUAD data and the GSVA algorithm were then used to identify the key modules most associated with immunogenic death, and differential genes were obtained by differential analysis of the TCGA-LUAD data. When we selected the intersection of immunogenic death marker genes and differential genes, we eventually found 167 genes involved in immunogenic death both in the single-cell transcriptome and in the bulk transcriptome. Afterwards, we performed regression analysis of these 167 genes using one-way COX to obtain genes related to OS, used these genes for consistent clustering to classify LUAD patients into two subtypes, and performed differential analysis of these two types to obtain differential genes. Finally, eight significant genes were screened using LASSO regression analysis and one-way COX risk regression analysis to create a new prognostic model. Significant prognostic differences were found between the two groups, demonstrating the independent predictive value of the ICD traits we created for LUAD.ROC curves proved the superior predictive efficacy of the ICD traits for patient prognosis. In addition, we compared our ICDRS with 11 other published articles, which showed good predictive efficacy of our ICDRS.

Then, to better understand TME may help to develop new therapies for LUAD or to repair TME to improve the effectiveness of immunotherapy. The composition of some immune cells differed between the two ICDRS groups. m0 and M1 macrophages were more common in the high ICDRS group, whereas Mast cells resting, Dendritic cells resting and T cells CD4 memory resting were more abundant in the low ICDRS group. Furthermore, based on the pathway enrichment results, we found that the low ICDRS group had stronger immune pathways, whereas the high ICDRS group contained more immunosuppressive cells and oncogenic signals, as well as tumor and metastasis-related signals, suggesting that the high ICDRS group exhibited immunosuppression and active tumor progression.

IPS data downloaded from TCIA can provide a predictive score for assessing a patient's response to immunotherapy 47-49. Higher IPS in the low ICDRS group suggests that patients with low ICDRS may have a more favorable response to ICI therapy. This study suggests that ICDRS, which have not been previously detected in LUAD, may correlate strongly with immune infiltration in LUAD, suggesting the potential relevance of ICDRS in assessing immunotherapy response. For patients with early-stage LUAD, surgical treatment, ablation, or liver transplantation are effective therapeutic modalities that can significantly improve patient survival time. For patients with advanced LUAD, systemic therapy is the only option to improve survival. In addition to the use of immunotherapy-related drugs, we also tend to use some chemotherapeutic drugs, of which in the vast majority of cases, the low ICDRS group will have a better therapeutic effect than the high ICDRS group, which will improve the survival time of patients with LUAD. The TIDE results also proved this point.

Based on these findings, we conclude that ICDRS is a good model for predicting survival time in LUAD patients and is closely related to the immune microenvironment. An in-depth study of ICDRS will be beneficial in treating patients with lung adenocarcinoma, thus improving the efficacy of immunotherapy. Next, eight genes comprise ICDRS: TPX2, RHOV, FAM83A, SFTPB, SERPIND1, FDCSP, CPS1, and KRT6A. we screened the key regulatory genes of ICDRS by ROC curve, TPX2 (Differentially Expressed In Cancerous And Non-Cancerous Lung Cells 2) TPX2 has been reported to mediate spindle filament assembly during mitosis and is associated with cell proliferation 50. TPX2 also promotes the EMT process and extracellular matrix degradation 51. FAM83A (family with sequence similarity 83, member A) is overexpressed in a variety of human tumors, including lung, breast, testicular, and bladder cancers, suggesting that FAM83A may play an oncogenic role in cancer development 52-54. FAM83A is involved in the regulation of a number of different tumor-associated signaling pathways, including EGFR, RAS/RAF/MEK/ERK and PI3K/AKT/mTOR pathways 55, 56. Ras homolog family member V (RHOV) plays an essential role in neurodevelopment and embryogenesis. 57, 58. RHOV has been reported to promote lung adenocarcinoma cell growth and metastasis through the JNK/c-Jun pathway 59. Although the regulatory roles of these genes have been studied in various cancers, few researchers have systematically evaluated their prognostic value in LUAD. Immunogenic death has been less studied in lung adenocarcinoma, and therefore we hope that the establishment of ICDRS will be used to improve the clinical management of lung adenocarcinoma patients.

This is despite the excellent ability of our constructed ICD signatures to identify patients' immune status and predict their prognosis. However, some limitations still need to be acknowledged in our follow-up study and find appropriate ways to address them. First, the TCGA-LUAD dataset we included was based on public database data, which may lead to bias in prediction results from the actual situation. Although we have taken several approaches to try to avoid this, more data from LUAD patients need to be collected to validate the utility of the model and the accuracy of the immunotherapy predictions, and we also need validation from external experiments.

## Conclusion

As we demonstrated for the first time, ICD modeling is a novel predictive biomarker and a possible therapeutic target for LUAD patients. ICDRS has better predictive efficacy compared to other published articles. In addition, the ICD model can characterize the immune environment of LUAD patients and appropriately estimate the prognosis of LUAD patients, which provides a new way of thinking for physicians to treat lung adenocarcinoma patients.

## Supplementary Material

Supplementary figures and tables.

## Figures and Tables

**Figure 8:**
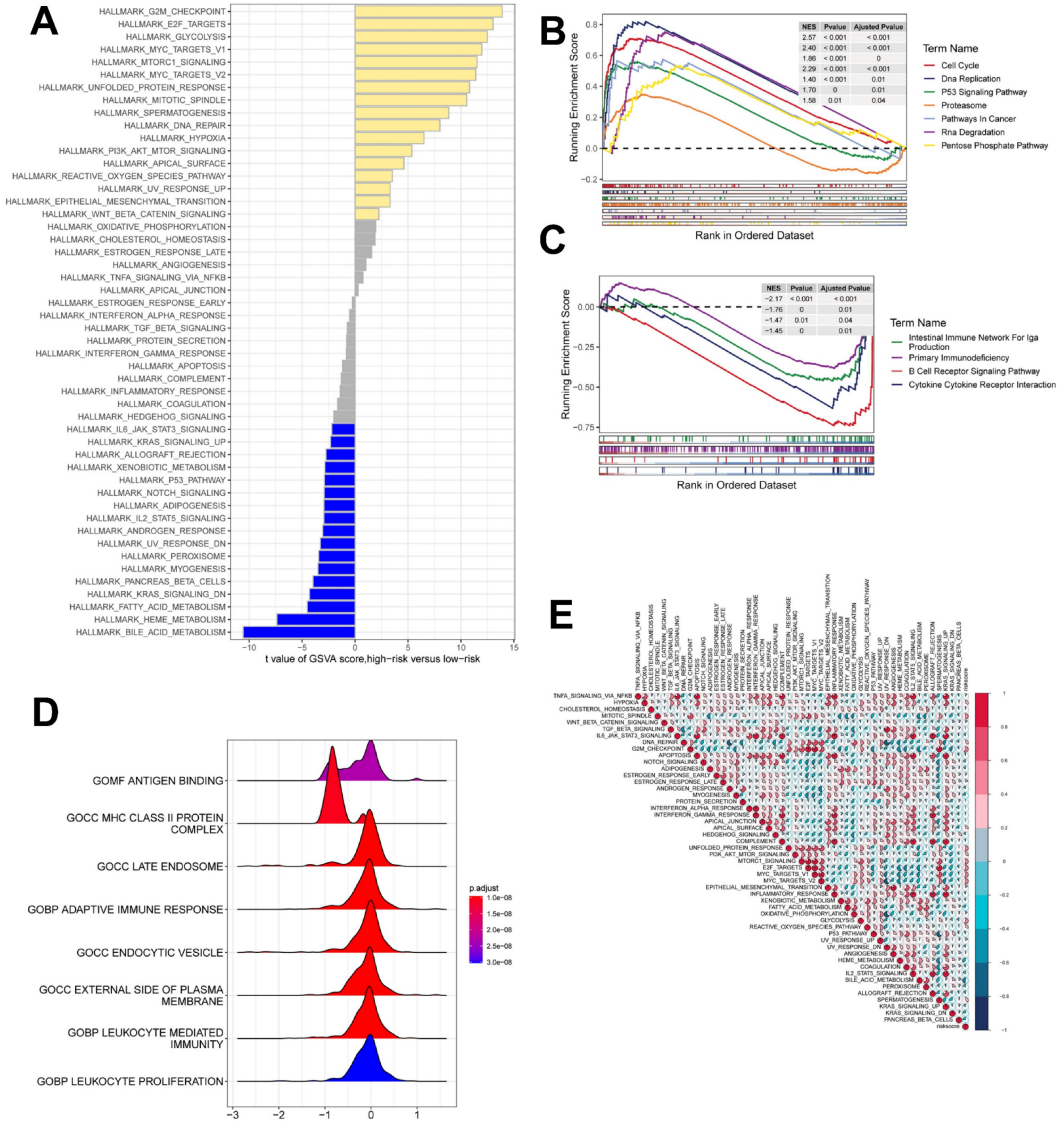
** gene set enrichment analysis. (A)** Differences in HALLMARK pathway activity between high and low risk groups with GSVA scores.** (B)** KEGG gene set enriched in the high ICDRS group.** (C)** KEGG gene set is enriched in the low ICDRS group.** (D)** Ridge diagram demonstrating the GO pathway between the two groups.** (E)** Correlation between risk scores and marker pathway activity for GSVA scores.

**Figure 11:**
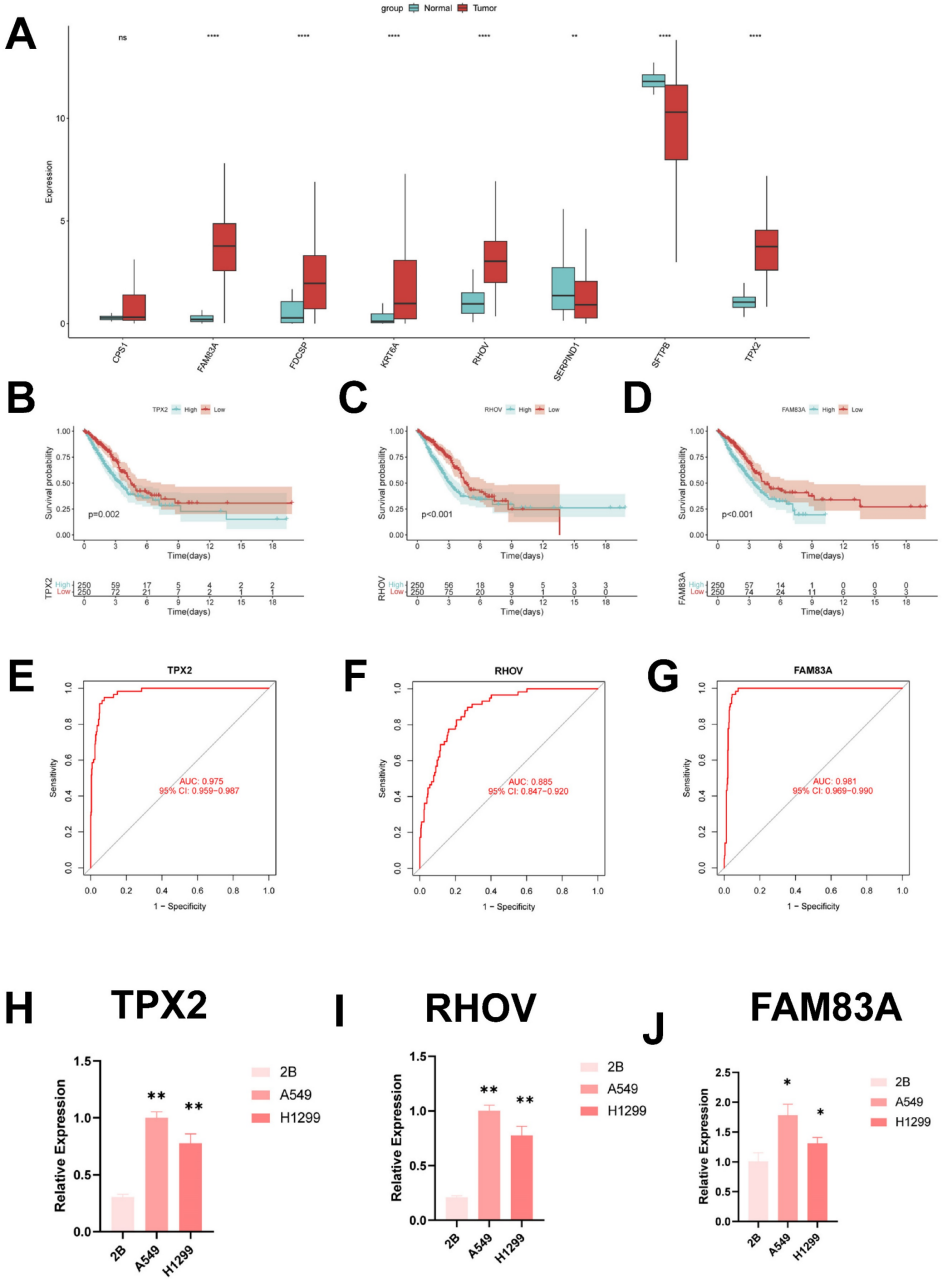
** Identification of key regulatory genes in the ICD model. (A)** Box-and-line plot demonstrating the expression of ICDRS genes in cancer and paracancer.** (B)** ROC diagnostic curve of TPX2.** (C)** ROC diagnostic curve of RHOV.** (D)** ROC diagnostic curve of FAM83A.** (E-G)** Demonstration of km curves of ICDRS genes.** (E)** TPX2.** (F)** RHOV.** (G)** FAM83A.** (H-J)** RT-qPCR demonstrating mRNA expression levels.** (H)** TPX2.** (I)** RHOV.** (J)** FAM83A.

**Table 1:** TCGA-LUAD Clinical characteristics.

Characteristics	High Risk (N=250)	Low Risk (N=250)	Overall (N=500)	P-value
**Age**				
<=65	124 (49.6%)	123 (49.2%)	247 (49.4%)	**0.321**
>65	126 (50.4%)	127 (50.8%)	253 (50.6%)	
**Gender**				
male	133 (53.2%)	97 (38.8%)	230 (46.0%)	**0.002**
female	117 (46.8%)	153 (61.2%)	270 (54.0%)	
**Stage**				
I	105 (42.0%)	163 (65.2%)	268 (53.6%)	**<0.001**
II	73 (29.2%)	46 (18.4%)	119 (23.8%)	
III	55 (22.0%)	25 (10.0%)	80 (16.0%)	
IV	16 (6.4%)	9 (3.6%)	25 (5.0%)	
unknown	1 (0.4%)	7 (2.8%)	8 (1.6%)	
**T stage**				
T1	60 (24.0%)	107 (42.8%)	167 (33.4%)	**0.00608**
T2	147 (58.8%)	120 (48.0%)	267 (53.4%)	
T3	31 (12.4%)	14 (5.6%)	45 (9.0%)	
T4	11 (4.4%)	7 (2.8%)	18 (3.6%)	
TX	1 (0.4%)	2 (0.8%)	3 (0.6%)	
**N stage**				
N0	143 (57.2%)	181 (72.4%)	324 (64.8%)	**0.00175**
N1	55 (22.0%)	39 (15.6%)	94 (18.8%)	
N2	47 (18.8%)	22 (8.8%)	69 (13.8%)	
N3	2 (0.8%)	0 (0.0%)	2 (0.4%)	
unknown	3 (1.2%)	8 (3.2%)	11 (2.2%)	
**M stage**				
M0	168 (67.2%)	164 (65.6%)	332 (66.4%)	**0.301**
M1	16 (6.4%)	8 (3.2%)	24 (4.8%)	
unknown	66 (26.4%)	78 (31.2%)	144 (28.8%)	
