# Supplementary material for: Identification of a novel immunogenic death-associated model for predicting the immune microenvironment in lung adenocarcinoma from single-cell and Bulk transcriptomes
Source: J Cancer. 2024 Aug 13;15(16):5165–82. doi: 10.7150/jca.98659 (PMC11375542; doi:10.7150/jca.98659)
Supplement: Supplementary file 1 — Supplementary figures and tables. [file jcav15p5165s1.zip › supplement.docx]

**Supplementary Figures:**


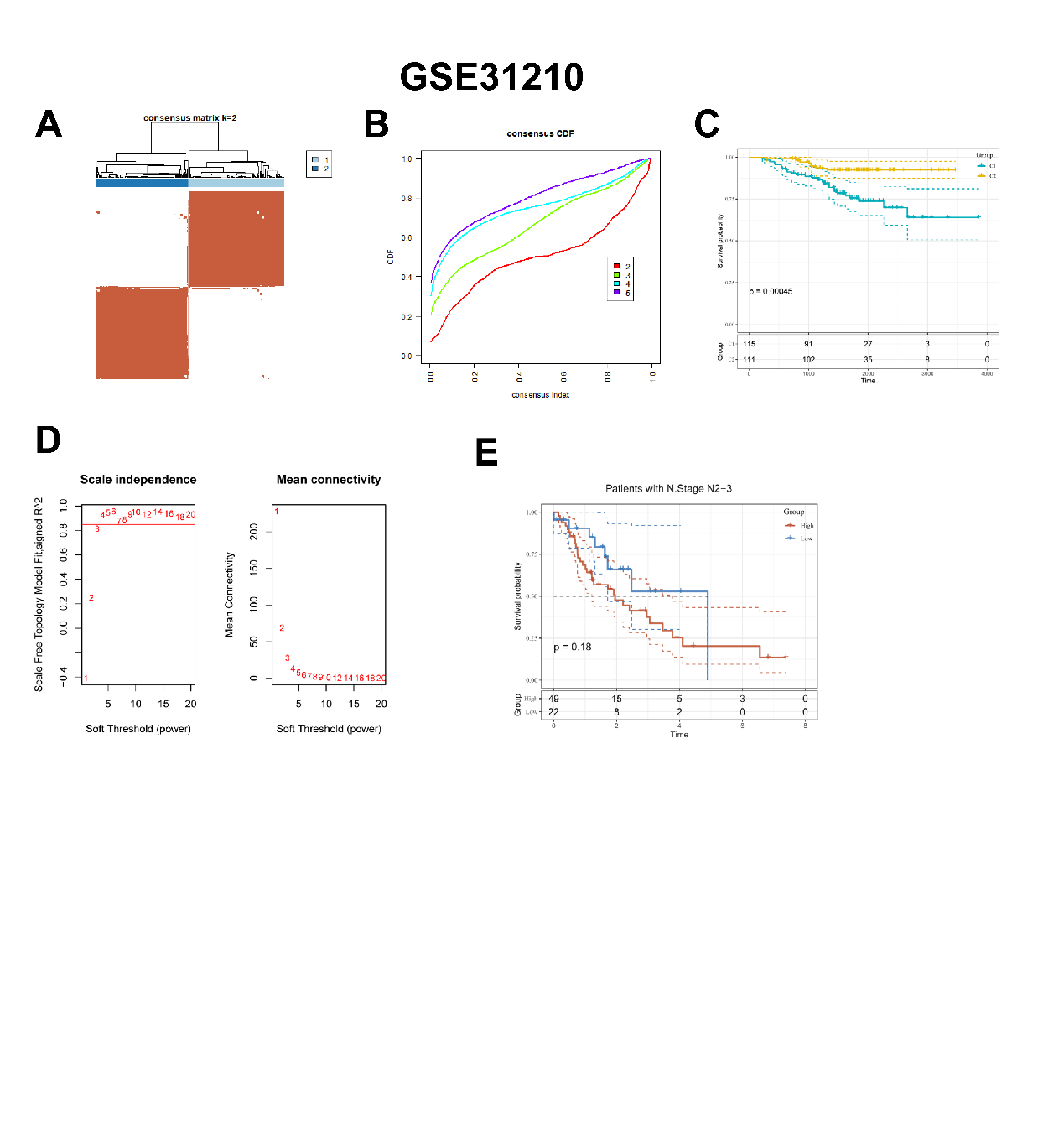


**Supplementary Figure.1**

**ConsensusClusterPlus and WGCNA identification of genes and km curves.**

(A)Consensus heatmap matrix and correlation region for two clusters (k = 2) (B) indicates that clustering results are best at K = 2. (C) Survival analysis indicates that C2 has a better prognosis. (D)WGCNA Screening Soft Queering. (E) km curves for N.Stage N2 and N3 patients.


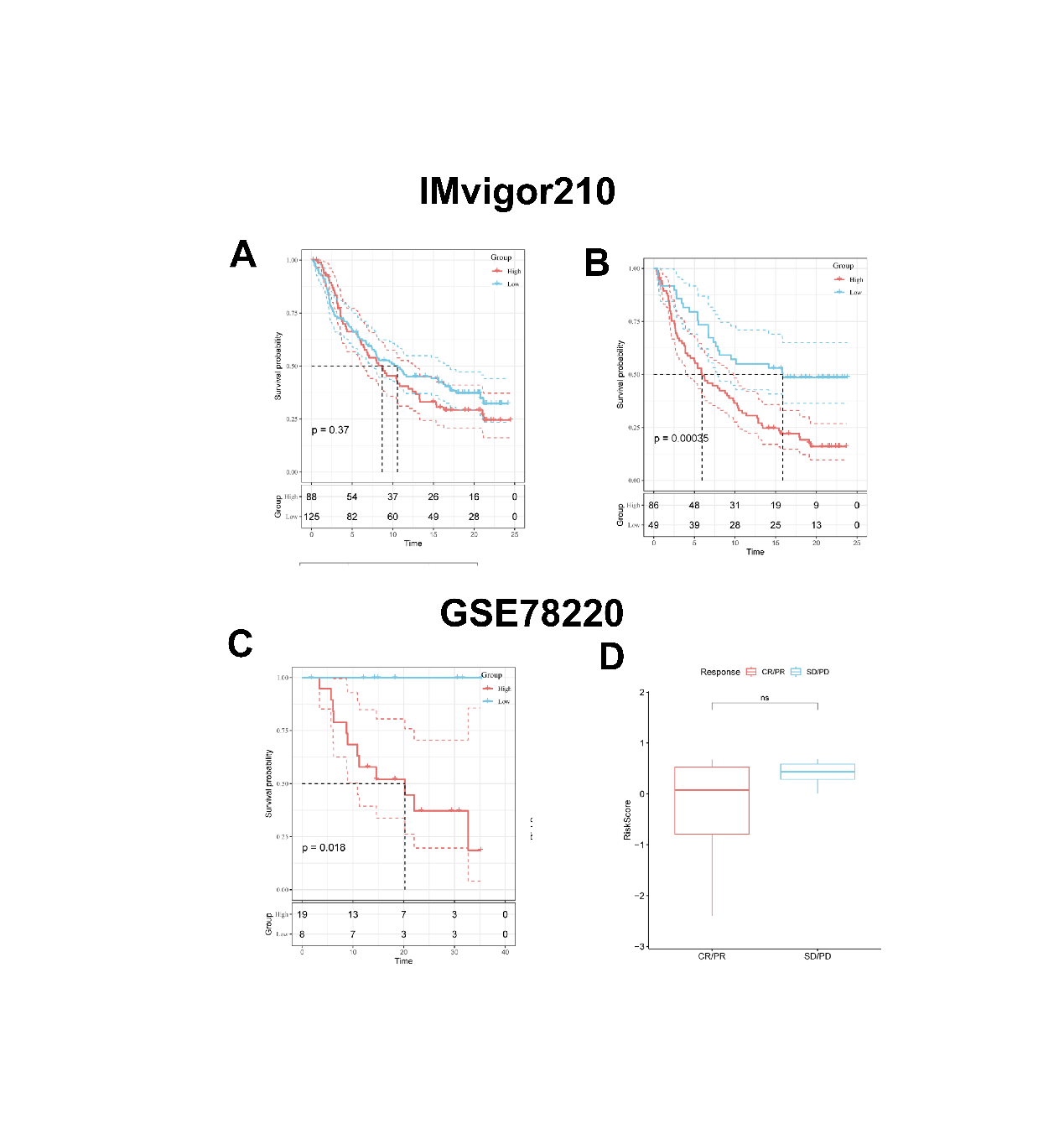


**Supplementary Figure.2**

**Immunotherapy for ICDRS.**

(A) IMvigor210 Stage I and II km curve. (B) IMvigor210 Stage III and IV km curve (C) The km curve of GSE78220. (D) Boxplot of GSE78220 Immunotherapy.


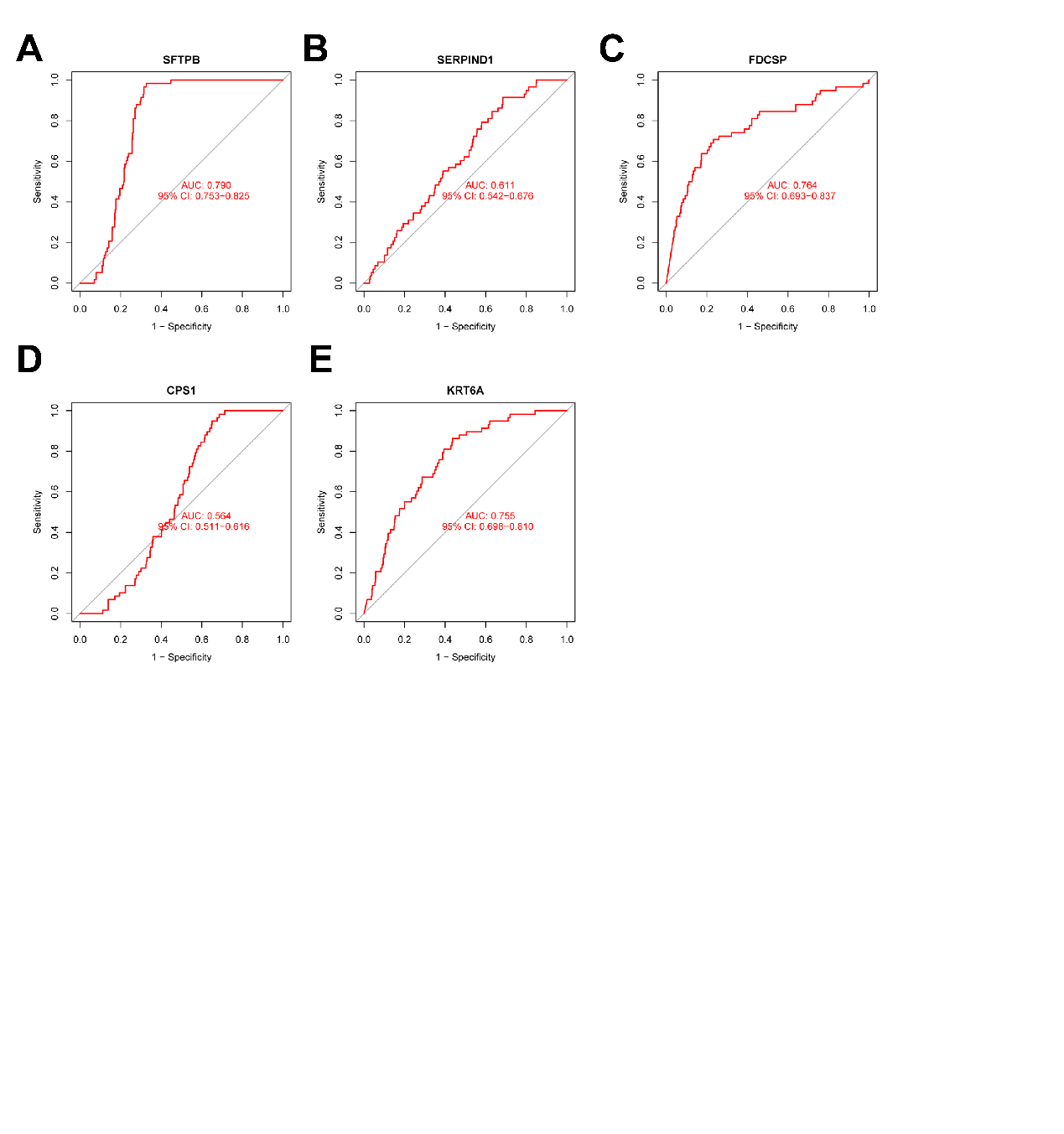


**Supplementary Figure.3**

**ROC diagnostic curve**

ROC diagnostic curve for the ICDRS gene including SFTPB(A),SERPIND1(B),FDCSP(C),CPS1(D),KRT6A(E).


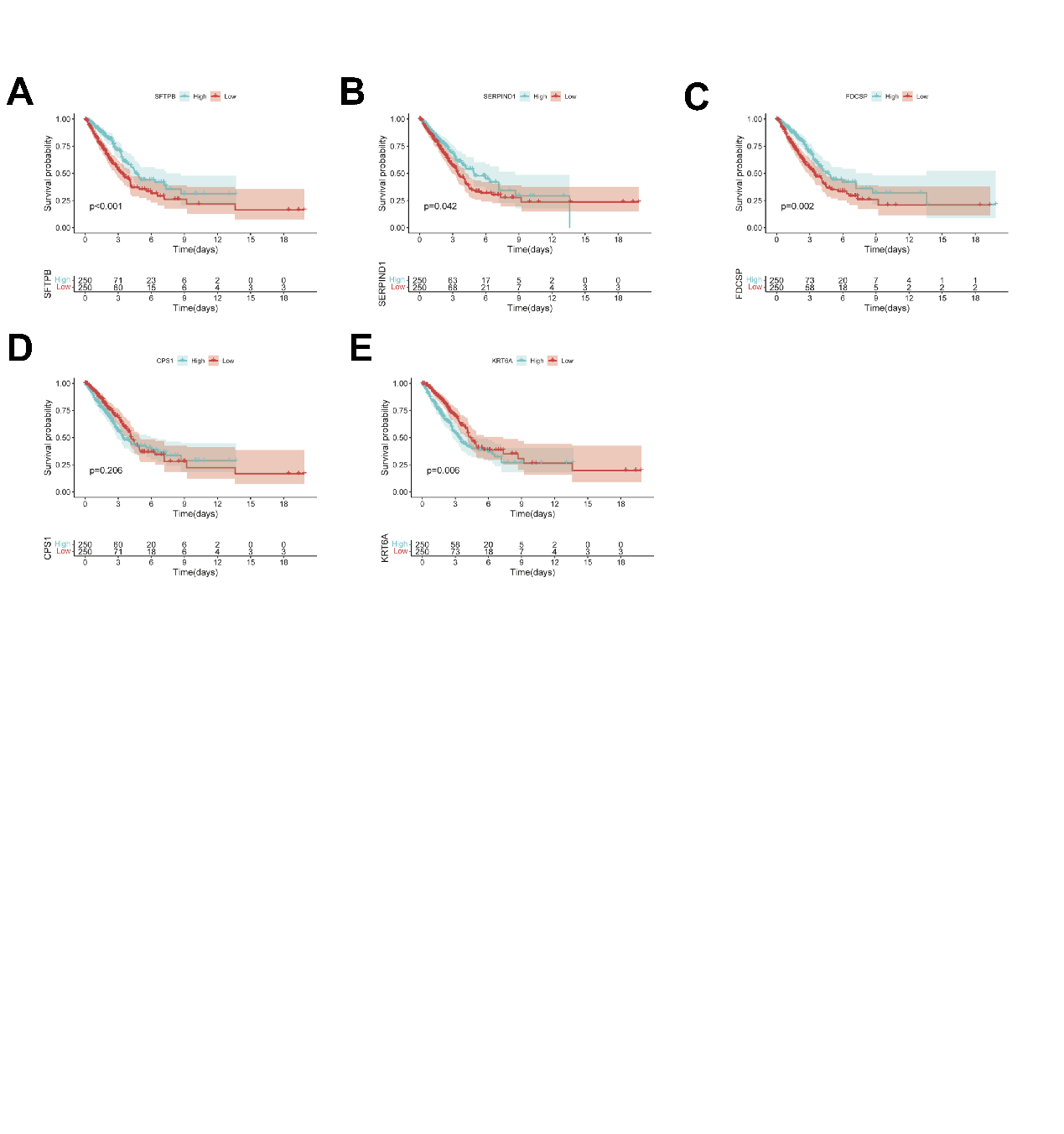


**Supplementary Figure.4**

**KM survival curves for the ICDRS gene**

KM survival curves for the ICDRS gene including SFTPB(A),SERPIND1(B),FDCSP(C),CPS1(D),KRT6A(E).
